# Emergence of Resistance in HIV-1 Integrase with Dolutegravir Treatment in a Pediatric Population from the IMPAACT P1093 Study

**DOI:** 10.1128/AAC.01645-21

**Published:** 2022-01-18

**Authors:** Cindy Vavro, Theodore Ruel, Andrew Wiznia, Nicole Montañez, Keith Nangle, Joseph Horton, Ann M. Buchanan, Eugene L. Stewart, Paul Palumbo

**Affiliations:** a ViiV Healthcare, Research Triangle Park, North Carolina, USA; b University of California San Francisco, San Francisco, California, USA; c Jacobi Medical Center, Albert Einstein College of Medicine, Bronx, New York, USA; d FHI 360, Durham, North Carolina, USA; e Parexel International, Durham, North Carolina, USA; f GlaxoSmithKline, Upper Providence, Pennsylvania, USA; g Geisel School of Medicine at Dartmouth, Lebanon, New Hampshire, USA

**Keywords:** HIV-1, integrase strand transfer inhibitor, dolutegravir, pediatric HIV

## Abstract

P1093 is a multicenter, open-label, phase I/II study of pharmacokinetics, safety, and tolerability of dolutegravir plus an optimized background regimen in pediatric participants aged 4 weeks to <18 years with HIV-1. Most participants were highly treatment experienced. We report the mechanisms of emergent integrase strand transfer inhibitor (INSTI) resistance among adolescents and children receiving dolutegravir. Plasma was collected at screening and near protocol-defined virologic failure (PDVF) for population-level and, for some samples, clonal-level integrase genotyping, phenotyping, and replication capacity. HIV-1 RNA was assessed in all available plasma samples. Phylogenetic analysis of clonal integrase sequences and homology modeling of HIV-1 intasome complexes containing resistance-associated substitutions were performed. Treatment-emergent INSTI resistance was detected in 8 participants who met PDVF criteria. The rare INSTI resistance-associated substitution G118R or R263K developed in 6 participants. The on-study secondary integrase substitution E157Q or L74I was observed in 2 participants. G118R reduced dolutegravir susceptibility and integrase replication capacity more than R263K and demonstrated greater reduction in susceptibility and integrase replication capacity when present with specific secondary integrase substitutions, including L74M, T66I, and E138E/K. Continuing evolution after R263K acquisition led to reduced dolutegravir susceptibility and integrase replication capacity. Structural examination revealed potential mechanisms for G118R- and R263K-mediated INSTI resistance. G118R and R263K INSTI resistance substitutions, which are distinct to second-generation INSTIs, were detected in adolescents and children with prior virologic failure who received dolutegravir. This study provides additional molecular and structural characterization of integrase to aid in the understanding of INSTI resistance mechanisms in antiretroviral-experienced populations. (This study has been registered at ClinicalTrials.gov under identifier NCT01302847.)

## INTRODUCTION

Adolescents and children with HIV-1 face multiple barriers to antiretroviral therapy (ART) adherence, including logistical challenges related to drug access and age-related factors, as well as issues with formulations, dosing frequency, pill or volume burden, toxicity, side effects, and others (1–3). As a result, there is a critical need to develop simple, safe, potent, and acceptable ART medications for adolescents and children with HIV-1.

Dolutegravir is a second-generation integrase strand transfer inhibitor (INSTI) that is potent and efficacious and has demonstrated strong efficacy, a high barrier to resistance, and a favorable safety and tolerability profile in treatment-naive and treatment-experienced adults with HIV-1 in phase III studies (4–6). Resistance to dolutegravir was not detected in studies of ART-naive and ART-experienced, virologically suppressed participants who failed treatment after starting a dolutegravir-based 3-drug regimen (6, 7). In INSTI-experienced participants who failed treatment after starting dolutegravir, no new INSTI resistance patterns were identified (8). However, in studies of dolutegravir treatment in ART-experienced, INSTI-naive individuals, there have been a small number of participants failing treatment with the uncommon INSTI resistance substitutions G118R and R263K (8, 9).

The International Maternal Pediatric Adolescent AIDS Clinical Trials Network (IMPAACT) P1093 study is evaluating the safety, tolerability, efficacy, and pharmacokinetics of dolutegravir in combination with an optimized background regimen for the treatment of HIV-1 in infants, children, and adolescents aged 4 weeks to <18 years in age-defined cohorts (10). Data from the P1093 study were instrumental in the recent U.S. Food and Drug Administration and European Medicines Agency approvals for dolutegravir dispersible tablets (DT) for children as young as 4 weeks of age and weighing at least 3 kg (11–14). Most adolescents and children enrolled in the P1093 study were highly treatment experienced and had evidence of virologic failure at screening (10, 15–17). A planned interim efficacy analysis before regulatory submission evaluated participants who completed or had the opportunity to complete through the week 24 visit. Here, we aimed to characterize the pathways of INSTI resistance that emerged among adolescents and children receiving dolutegravir who met protocol-defined virologic failure (PDVF) criteria in the P1093 study as part of a planned interim analysis.

## RESULTS

### Baseline characteristics.

Of 142 participants in the P1093 study included in this analysis, 36 (25%) met PDVF criteria through the data cutoff and had genotypic analysis performed. Of those meeting PDVF criteria, 8 (22%) participants across the treatment cohorts had on-treatment observations of resistance-associated integrase substitutions at or near PDVF (Table 1). All 8 participants were nucleoside reverse transcriptase inhibitor (NRTI) and protease inhibitor (PI) experienced, and 3 had prior nonnucleoside reverse transcriptase inhibitor (NNRTI) exposure. Optimized background regimens were 2 NRTIs (*n* = 5), 2 NRTIs + 1 NNRTI (*n* = 1), 2 NRTIs + 1 boosted PI (*n* = 1), and 1 NRTI + 1 NNRTI + 1 boosted PI (*n* = 1). Six of the 8 participants had optimized background regimens that consisted of ≥1 antiretroviral agent used in a prior ART regimen. Based on discussions with the study personnel, each of the 8 participants was judged to be nonadherent to the study regimen leading up to the PDVF visit. HIV-1 subtypes were B (*n* = 5), C (*n* = 1), and CRF01_AE (*n* = 2).

**TABLE 1:** Baseline characteristics^*a*^

Patient	Cohort	Age (yr)	HIV-1 subtype	HIV-1 RNA level (copies/mL)	Prior ART agent(s)				Optimized background ART
					Duration (mo)	NRTI	NNRTI	PI	
1	I	12	B	7,739	144	ZDV, D4T, 3TC, ABC		NFV, LPV, RTV	FTC, TDF
2	I	16	B	17,996	152	3TC, ZDV, D4T, DDI, FTC, TDF		RTV, ATV	EFV, FTC, TDF
3	IIB	7	B	96,369	85	ZDV, ABC, 3TC	NVP	LPV, RTV	3TC, ZDV
4	III	5	B	1,605,957	13	3TC, ZDV		LPV, RTV	3TC, ZDV
5	III-DT	2	C	30,531	28	ABC, 3TC		LPV, RTV	3TC, ZDV
6	IV	1	CRF01_AE	594	9	D4T, 3TC	NVP	LPV, RTV	D4T, 3TC
7	IIA	11	B	890	132	ZDV, 3TC, D4T, DDI, ABC	EFV	NFV, LPV, RTV	FTC, TDF, RTV, ATV
8	III-DT	5	CRF01_AE	846,872	69	3TC, ZDV		LPV, RTV	3TC, EFV, RTV, DRV

a ABC, abacavir; ART, antiretroviral therapy; ATV, atazanavir; DDI, didanosine; DRV, darunavir; DT, dispersible tablet; D4T, stavudine; EFV, efavirenz; FTC, emtricitabine; LPV, lopinavir; NFV, nelfinavir; NNRTI, nonnucleoside reverse transcriptase inhibitor; NRTI, nucleoside reverse transcriptase inhibitor; NVP, nevirapine; PI, protease inhibitor; RTV, ritonavir; 3TC, lamivudine; TDF, tenofovir disoproxil fumarate; ZDV, zidovudine.

### Clinical outcomes.

**FIG 1:**
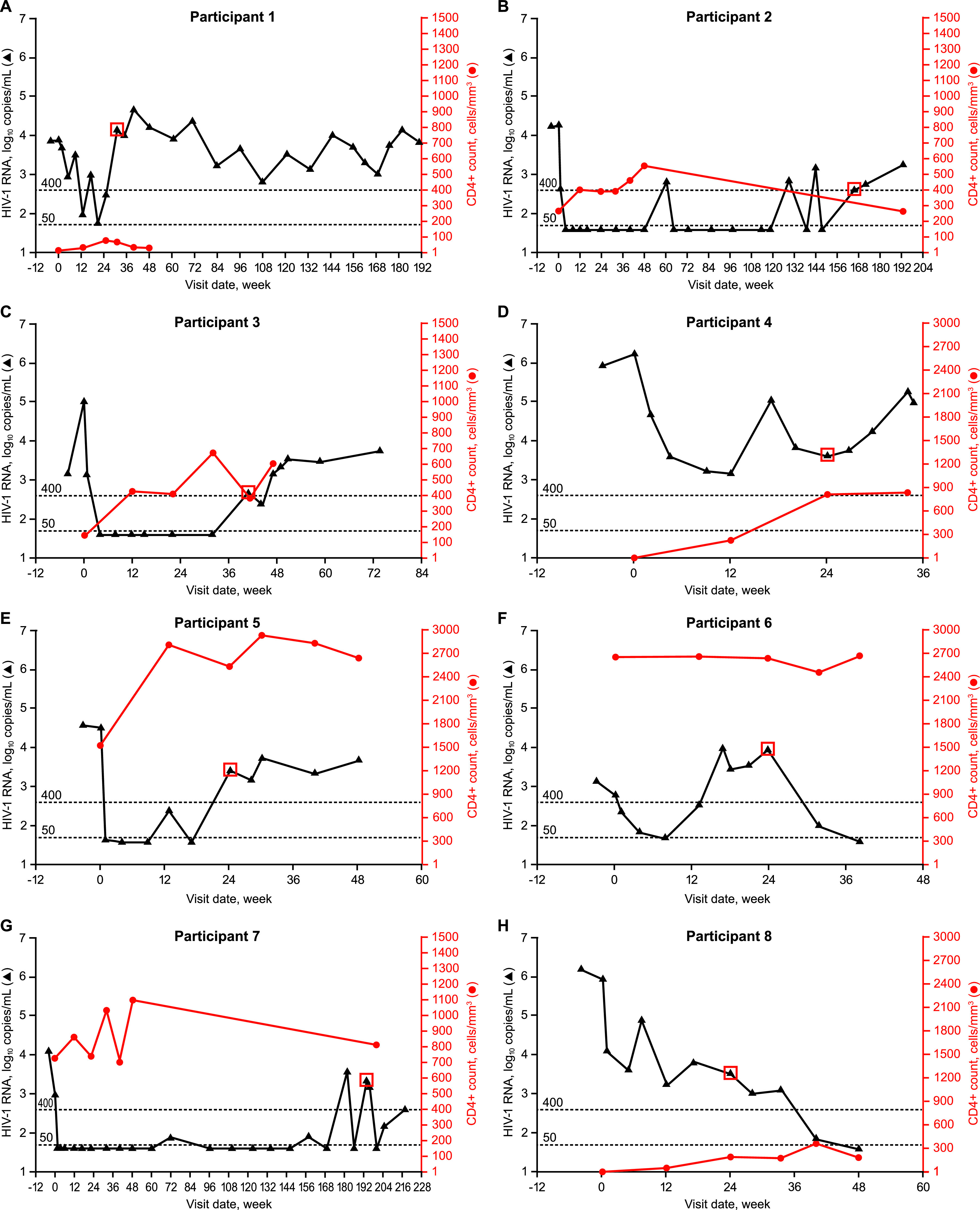
HIV-1 RNA and CD4^+^ cell count over time in participants with PDVF and treatment-emergent resistance-associated substitutions in integrase. Red boxes denote HIV-1 RNA at the week each participant met PDVF criteria. Solid and dashed lines indicate HIV-1 RNA levels of 400 and 50 copies/mL, respectively.

Of the 8 participants with detected treatment-emergent resistance-associated integrase substitutions, PDVF criteria were met at week 24 (*n* = 4) and weeks 32, 40, 132, and 192 (*n* = 1 each) (Fig. 1). Before PDVF occurred, 6 participants had achieved HIV-1 RNA levels of <400 copies/mL, 4 of whom achieved levels of <50 copies/mL for several weeks. Of the 6 participants with available CD4^+^ cell count data at the time of PDVF, 5 showed an increase from baseline (median [range], 240 [54 to 1,012] cells/mm^3^), consistent with the observed initial decline in HIV-1 RNA. After PDVF occurred, participant 6 resuppressed (HIV-1 RNA <50 copies/mL) with reoptimization of the background regimen. The remaining 7 participants remained in the study after PDVF for 32 to 216 weeks.

### Population genotypic and phenotypic analysis.

Of the 8 participants with resistance-associated integrase substitutions, 6 had treatment-emergent rare INSTI-associated substitutions G118R (*n* = 5) or R263K/R (*n* = 1) during the course of treatment (Table 2). In 2 participants, a single INSTI-associated polymorphic substitution of E157Q or L74I (*n* = 1 each) was identified during the study, but given that no pretreatment samples from either participant were available for integrase assessment, it was not possible to determine if emergence had occurred during the study period. Four of 5 participants with G118R had 1 additional treatment-emergent INSTI-associated substitution of L74M, E138E/K, E92E/Q, or T66I, respectively. Of 5 participants with available drug sensitivity data on or near PDVF, 4 (all of whom had G118R substitutions) demonstrated *in vitro* resistance to dolutegravir (Table 2). Treatment-emergent protease substitutions did not occur in any participant at PDVF; only 2 participants were taking a PI as part of their optimized background regimen. Three participants had additional treatment-emergent resistance-associated reverse transcriptase substitutions of T215F/L, T215F/I/S/T and M230I/M, or M184V (*n* = 1 each) at PDVF. One of the 5 participants with detection of G118R (participant 6) was able to fully suppress (HIV-1 RNA level, <50 copies/mL) after confirmation of PDVF with the addition of lopinavir/ritonavir to the study regimen.

**TABLE 2:** Genotype at baseline and PDVF in participants with INSTI resistance-associated substitutions^*a*^

Patient	Wk of PDVF^*b*^	Integrase			Protease		Reverse transcriptase	
		Baseline	PDVF^*c*^	Fold change in dolutegravir sensitivity^*d*^	Baseline	PDVF^*b*^	Baseline	PDVF^*c*^
1	32	L74L/M	**R263K/R** ^*e*^	1.1^*f*^	I84I/V			
2	168		**L74M**, **G118R**	25.1			M41L, T215L	M41L, **T215F/L**
3	40	V151I	**G118R**, **E138E/K**, V151I	5.9			K103S, V106I/V, V179I/V, M184M/V, G190A, Y318F	K103S, M184V, G190A
4	24		**E92E/Q**, **G118G/R**	NA			T69A/D/N/T	**M184V**
5	24	L74I	L74I, **G118R**	9.6^*g*^			M184V, H221Y	M184V, H221Y
6	24	L74I	**T66I**, L74I, **G118R**	19.3	K20R	K20R	V179I, M184V, K238R	V179I, M184V, K238R
7	192	NA	**E157Q**	NA			V179I	V179I
8	24	NA	**L74I**	NA	L10I/L, K20K/R, L33F, M46I, I50V, I54V, T74P, V82A	L10I/L, K20K/R, L33F/L, M46I/M, I50I/V, I54I/V, T74P/T, V82A/V	M41L, D67N, T69N/T, K70R, L74I, A98G, M184V, T215F, K219Q, K238R	M41L/M, D67D/N, T69N/T, K70K/R, L74I/L, A98A/G, M184M/V, **T215F/I/S/T**, K219Q, **M230I/M**, K238R

a INSTI, integrase strand transfer inhibitor; NA, not available; PDVF, protocol-defined virologic failure.

b Testing for resistance took place at the next available time point after PDVF for participant 1 (week 36), participant 2 (week 192), and participant 3 (week 52).

c Treatment-emergent substitutions are in bold.

d Fold change in dolutegravir susceptibility at study visits after meeting PDVF criteria except where noted. The clinical cutoff for dolutegravir is 4.0.

e Genotypes at weeks 136 and 168 were E138A/E/K/T, S147G/S, and R263K and E138T, S147G, and R263K, respectively.

f The fold change in dolutegravir susceptibility at weeks 136 and 168 was 5.0 and 5.1, respectively. The fold change in dolutegravir susceptibility at baseline was 1.04.

g The fold change in dolutegravir susceptibility at baseline was 0.62.

Participant 1 remained in the study after meeting PDVF criteria at week 32 and had additional genotypic and phenotypic data available at weeks 136 and 168 (Table 2). Testing at week 36 showed that virus from participant 1 had treatment-emergent R263K/R but was susceptible to dolutegravir (fold change, 1.1). Additional integrase substitutions accumulated at weeks 136 and 168, with genotyping results of E138A/E/K/T, S147G/S, and R263K and E138T, S147G, and R263K, respectively. Participant 1 also demonstrated *in vitro* resistance to dolutegravir at weeks 136 and 168 (fold change, 5.0 and 5.1, respectively).

For the 6 participants with emergent R263K or G118R, prior ART and optimized background regimen summaries and longitudinal reverse transcriptase and protease genotypic data may provide additional details on the emergence of resistance to dolutegravir. Selection of 1 fully active drug based on a genotype obtained at study screening satisfied the study’s inclusion requirement for the optimized background regimen. However, data from Tables 1 and 2 show that optimized background regimen selection for these participants would be predicted to have limited activity due to preexisting resistance or evolution of resistance to components of the on-study optimized background regimen.

### Clonal genotypic and phylogenetic analysis of HIV integrase.

Clonal analyses of integrase genotypes were performed on available plasma samples from 3 participants with rare INSTI-associated substitutions (Table 3). Testing was performed on plasma samples collected on or near PDVF. Individual clonal sequences show linkage of identified INSTI-associated substitutions. Clones from participant 2 with G118R and either L74M or L74M/V75A treatment-emergent integrase substitutions exhibited increased fold changes in dolutegravir susceptibility and decreased integrase replication capacity compared with wild-type clones at baseline. Similar results were observed for clones from participant 3, who had only treatment-emergent G118R compared with clones analyzed at baseline. Treatment-emergent R263K alone in clones at week 36 from participant 1 did not affect fold change in dolutegravir susceptibility or integrase replication capacity compared with wild-type clones at baseline. However, week 136 clones from participant 1 with treatment-emergent R263K plus additional integrase substitutions resulted in increased fold change in dolutegravir susceptibility and decreased integrase replication capacity compared with clones at baseline and week 36.

**TABLE 3:** Clonal integrase genotypes and drug sensitivity in selected participants with INSTI resistance-associated substitutions

Patient	Study visit^*a*^	Integrase substitution(s)^*b*^	No. of clones	Median fold change in drug sensitivity			Median integrase replication capacity (%)
				Dolutegravir^*c*^	Elvitegravir^*d*^	Raltegravir^*e*^	
1	Pretreatment	L74V	4	0.97	1.28	1.10	95
		L74I	1	0.97	1.22	0.90	29
		L74L	3	1.16	1.03	1.28	81
	Wk 36	**R263K**	4	2.0	2.3	1.37	97
		V201I	3	1.19	1.11	1.11	92
		V201V, R263R	1	1.26	1.31	1.15	128
	Wk 136	*A49G*, *M50V*, V201I, **R263K**	12	4.17	3.6	1.76	49
		*A49G*, *M50V*, **E138T**, **S147G**, V201I, **R263K**	4	6.33	4.83	2.22	28
2	Pretreatment	Wild-type	8	0.9	1.8	1.0	87.5
	Wk 192	**L74M**, **G118R**	15	22	31	36	5.5
		**L74M**, V75A, **G118R**	1	52	76	> MAX	0.28
3	Pretreatment	V151I	8	0.9	1.1	0.8	147
	Wk 52	V151I, **G118R**	16	9.6	6.0	12.5	17.5

a Testing for resistance took place at the next available time point after PDVF for participant 1 (week 36), participant 2 (week 192), and participant 3 (week 52).

b INSTI resistance-associated substitutions are in bold (27). Additional integrase substitutions of interest are in italics.

c The clinical cutoff for dolutegravir is 4.0.

d The biological cutoff for elvitegravir is 2.5.

e The biological cutoff for raltegravir is 1.5.

**FIG 2:**
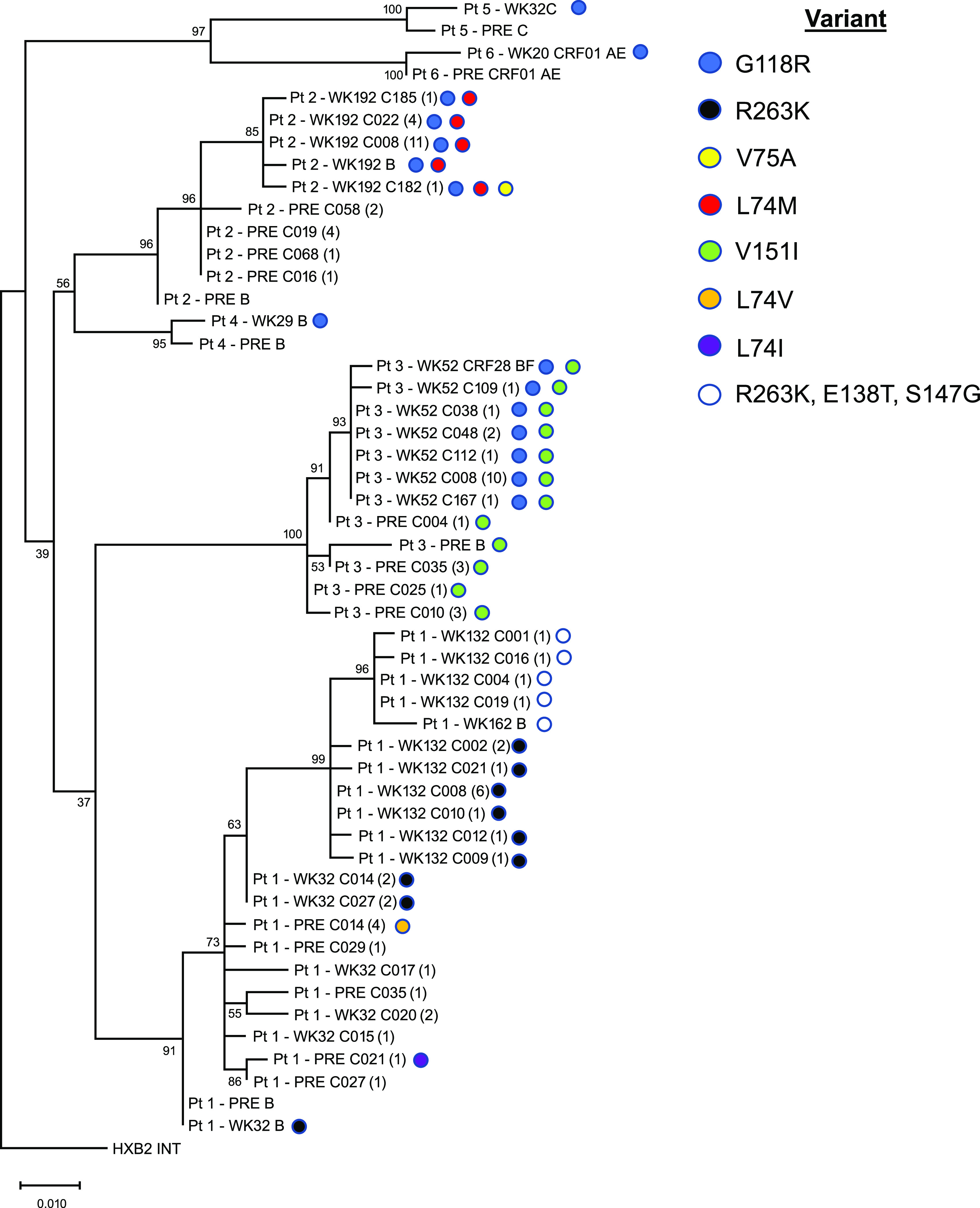
Phylogenetic analysis of clonal and population integrase amino acid sequences from participants 1 through 6. Bootstrap confidence levels are indicated for each sequence cluster. For population sequences, the naming convention is as follows: participant number, time point in the study, HIV-1 subtype. For clonal sequences, the naming convention is as follows: participant number, time point in the study, C clone identification number (number of clones with identical sequences).

Results from the phylogenetic analysis of sequences from available plasma samples demonstrated common ancestry for each participant sequence cluster (Fig. 2). Sequences from participant 1 at pretreatment and week 36 clustered together, with none of the pretreatment and 4 of the 8 week 36 clones containing R263K. All week 136 clonal sequences and 1 week 168 population sequence contained R263K and clustered together (bootstrap = 99%). A subcluster of sequences containing E138T, S147G, and R263K showed greater evolutionary distance (bootstrap = 96%). Analysis for participant 2 showed that all week 192 clonal and population sequences collected after PDVF (week 168) clustered together (bootstrap = 85%); each sequence contained G118R, and 11 of 17 clones were identical at the nucleotide level. All week 52 clonal and population sequences from participant 3 collected 12 weeks after PDVF (week 40) clustered together (bootstrap = 93%), with 10 of 16 clones being identical at the nucleotide level. All sequences from this cluster contained G118R and V151I; V151I was also present in all pretreatment sequences in this participant. As expected, sequences from participants 5 and 6 who had no further clonal analysis data available and HIV-1 subtypes C and CRF01_AE, respectively, formed clusters separately from each other and the subtype B sequences.

### HIV integrase structural analysis.

**FIG 3:**
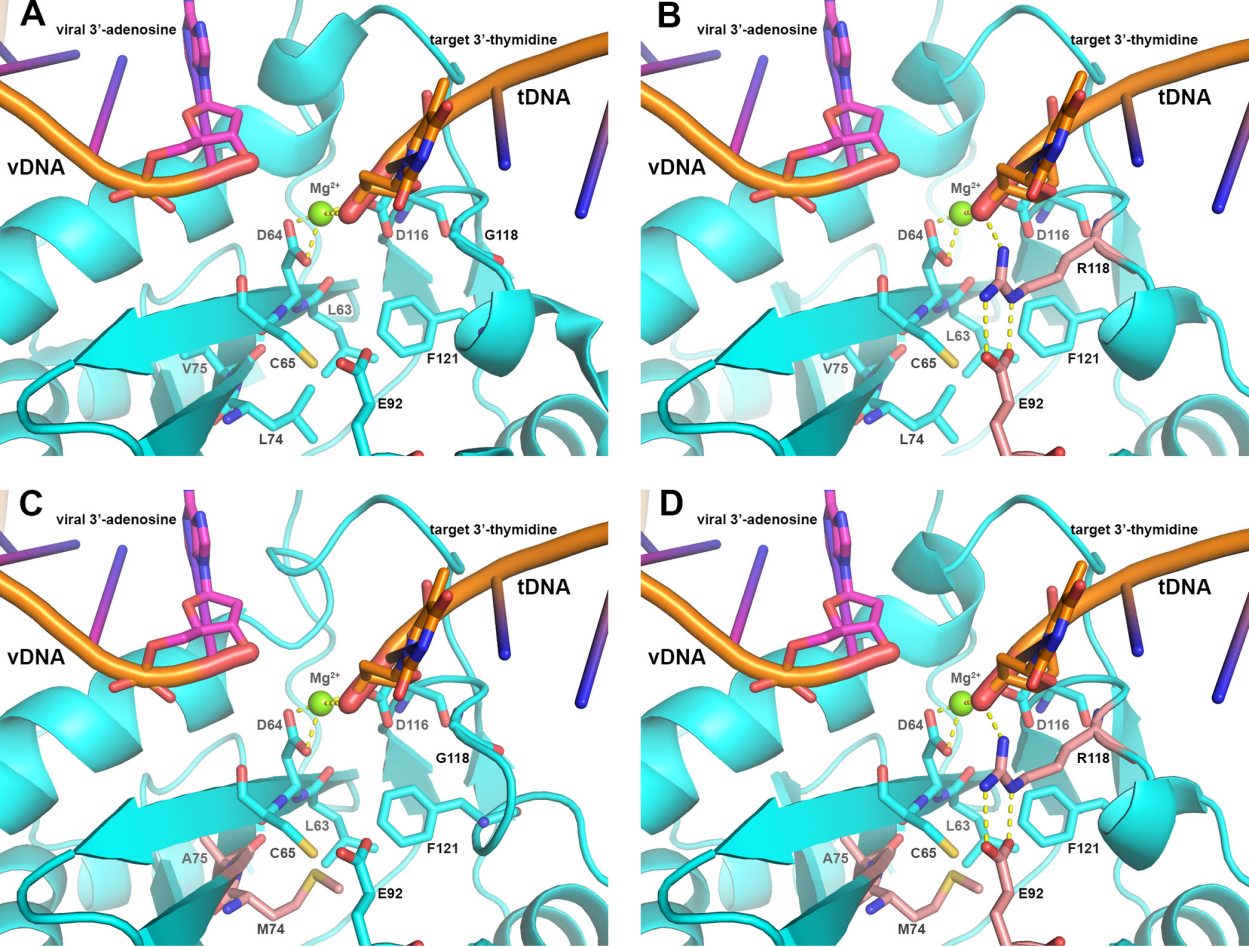
Catalytic site of wild-type or mutant HIV-1 integrase in the intasome complex. HIV-1 integrase is shown in cartoon and colored cyan. Selected catalytic site amino acid residues are displayed as sticks and colored cyan unless otherwise designated. Both the vDNA and tDNA substrates are shown in cartoon and colored orange. The terminal viral 3′ adenosine and target 3′ thymidine are displayed in cartoon and colored magenta and orange, respectively. The catalytic magnesium (Mg^2+^) is displayed in ball-and-stick format and colored green. Hydrogen bonds are depicted as yellow dashed lines. (A) View of the wild-type HIV-1 catalytic site illustrating the vDNA/tDNA interface and the mechanism of the integration process facilitated by D64, D116, and the Mg^2+^. (B) Identical view of the G118R HIV-1 catalytic site illustrating the hydrogen bonding complex formed among the terminal tDNA, R118 (in pink), and E92 (in pink) with the addition of the G118R resistance mutant. (C) Identical view of the L74M/V75A HIV-1 catalytic site illustrating the location of M74 (in pink) and A75 (in pink) relative to the nearby catalytic site residues L63, C65, and F121. (D) Identical view of the L74M/V75A/G118R HIV-1 catalytic site illustrating the location of M74 (in pink) and A75 (in pink) relative to R118 (in pink) and E92 (in pink). tDNA, host target DNA; vDNA, viral DNA.

To further explore the impact of the observed integrase substitutions seen in this study, homology models of the HIV-1 integrase active site were examined (Fig. 3; see Text S1 and Movie S1 in the supplemental material for additional details). Structural analysis of wild-type HIV-1 integrase bound with viral DNA (vDNA) demonstrated that R263 forms multiple hydrogen bonds among the catalytic loop, including a dual hydrogen bond with N144 and with both the 3′ and 5′ termini of the vDNA (9). In the integrase R263K mutant, all but one hydrogen bond with the substrate and catalytic loop were eliminated, resulting in a differential geometry of the catalytic site relative to wild-type HIV-1 integrase. Homology models of the HIV-1 integrase in the context of the intasome complex reveal that the L74M, V75A, and G118R resistance mutants are clustered near the HIV-1 integrase catalytic site. Specifically, these residues are in close proximity to the catalytic site residues L63, D64, C65, E92, and F121. Those proximal residues, along with the 3 resistance mutations, are located on or near the HIV-1 integrase host target DNA (tDNA) catalytic loop. Additionally, the L74M V75A G118R triple mutant HIV-1 integrase model shows a complex network of hydrogen bonds among R118, E92, and the tDNA terminus, as has been previously observed in G118R mutants (9, 18, 19).

## DISCUSSION

This report characterizes the development of INSTI resistance among 8 pediatric participants in the P1093 study who acquired resistance-associated integrase substitutions while receiving dolutegravir. In 6 of the 8 participants, the INSTI substitution G118R or R263K emerged during dolutegravir treatment. HIV-1 clones with either of these INSTI substitutions impacted fold changes in dolutegravir susceptibility; however, the degree of reduced susceptibility was influenced by the presence of ≥1 secondary INSTI substitutions. Clonal data showed these secondary substitutions to be linked on the same genome with either G118R or R263K. Modeling data described the impact of these linked INSTI substitutions on the integrase structure and their ability to constrict the catalytic pocket and reduce dolutegravir binding. Clinically, each participant showed an ability to initially suppress HIV-1 on dolutegravir treatment, but the degree of suppression before PDVF varied across the 8 participants; adherence (per the site’s investigator) was decreased for each participant leading up to the detection of INSTI resistance. While this investigation provided additional knowledge on observed INSTI resistance patterns, there was not enough information available to identify a clinical or virological factor that would predict the emergence of these INSTI resistance substitutions in children.

Emergent INSTI resistance has been previously reported among pediatric patients aged 4 weeks to <19 years who had virologic failure after treatment with the first-generation INSTI raltegravir in the P1066 study. Raltegravir resistance-associated substitutions developed in 15 of 40 participants with virologic failure by week 48 and in 19 of 60 by week 240 (3, 20, 21).

The INSTI resistance substitutions observed in the P1093 study were similar to those observed in ART-experienced adults (9). In the larger adult phase III studies DAWNING and SAILING, emergence of G118R and/or R263K with dolutegravir occurred in <2% of participants (9, 22). Adults with G118R and/or R263K substitutions exhibited suppressed or declining viral loads before meeting PDVF criteria, similar to observations in the P1093 study. In observations from adults, the reported degree of reduced dolutegravir susceptibility when either INSTI substitution was present was similar to observations from the P1093 study. In addition, a recent meta-analysis of 11 studies enrolling >1,100 INSTI-naive adults also reported 19 cases with development of G118R (*n* = 6) and/or R263K (*n* = 13) while on a dolutegravir-based regimen (8). This meta-analysis reported that G118R was observed most often in dolutegravir monotherapy studies (8). Importantly, there have been no clinical observations of G118R or R263K integrase substitutions in adult participants experiencing virologic failure while receiving fixed-dose combinations of dolutegravir, such as abacavir-dolutegravir-lamivudine or dolutegravir-lamivudine, or in those who are virologically suppressed and starting a dolutegravir-based 3-drug regimen (8, 23, 24).

In contrast to adult efficacy trials, the P1093 study had primary objectives of safety and pharmacokinetics to gain regulatory approval, more limitations on clinical trial enrollment, and no comparator group. While the P1093 study recruited mostly ART-experienced participants, no ART-naive participants who enrolled in P1093 and started dolutegravir during the study experienced PDVF with emergent INSTI resistance. Each participant with emergent G118R or R263K was receiving a genotype-derived optimized background regimen of ≥2 agents, but 5 of the 6 participants with emergence of these INSTI substitutions were recycling agents from their ART history; the sixth participant had emtricitabine as part of the optimized background regimen after prior ART that included lamivudine. No participant in the P1093 study received dolutegravir as part of a fixed-dose combination. Given that the majority of the study population was highly treatment experienced, there were limited options for subsequent regimens in the event of virologic failure, and drug availability varied between countries. As a result, participants were permitted to remain on study treatment after meeting PDVF criteria despite resistance development, potentially contributing to evolution of resistance observed in this study compared with the adult study. Collectively, these results point to the fundamental challenges involved in conducting clinical studies in pediatric populations with HIV-1 and contribute to the limitations of this study.

Adherence to medication is critical for ART efficacy in any population; children experience multiple unique barriers to complete ART adherence (2). However, adherence is inherently difficult to study rigorously in pediatric populations. Although lack of adherence was reported for each participant with INSTI resistance detection in the P1093 study, there was no specific adherence information available for each component of the regimen. Thus, it is possible that a participant was adherent to all elements of a treatment regimen or only a portion of the regimen, which might be expected to result in additional resistance. Indeed, for participant 1, who had emergent R263K, there were reports of nonadherence to the optimized background regimen and only sporadic adherence to dolutegravir leading up to and after PDVF. However, it is important to note that this result was determined through participant self-reporting in the P1093 study. Together, data from P1093 underscore the lack of sensitivity of the study’s 3-day recall measure of adherence. For future pediatric phase II studies, hair levels or the use of dried blood spots could provide additional data for individual drug levels to gain information about adherence (25, 26).

Of the 6 participants with PDVF and resistance to dolutegravir, 5 developed G118R. The clinical factors noted above describe a clinical setting in which antiretroviral resistance may generally develop and may also specifically address the high number of participants with this uncommon integrase substitution. All 5 participants were receiving optimized background regimens that could be considered weaker (due to prior use or resistance), and each struggled with adherence. Together, these 2 factors could provide a setting of functional monotherapy with dolutegravir and a higher risk of resistance development. As noted above, G118R has been most frequently observed in the setting of dolutegravir monotherapy (8).

Population and clonal integrase phenotypic analyses indicated that accumulation of integrase substitutions is associated with decreased dolutegravir susceptibility. G118R had a greater impact on reduced dolutegravir susceptibility than R263K. Additionally, integrase replication capacity was reduced in viral clones with G118R compared with those with R263K. Specific secondary integrase substitutions also impacted the effect of G118R and R263K on dolutegravir susceptibility. Treatment-emergent G118R in combination with either L74M or T66I was associated with reduced dolutegravir susceptibility compared with G118R alone or with E138E/K. Clonal phenotyping demonstrated that a viral clone with G118R, L74M, and V75A had reduced susceptibility to dolutegravir, elvitegravir, and raltegravir compared with clones with only G118R and L74M. Consistent with these results, the clone with G118R, L74M, and V75A exhibited reduced integrase replication capacity compared with the G118R L74M clones. Thus, V75A and the known INSTI resistance-associated substitutions L74M and T66I may cooperate with G118R to exacerbate INSTI resistance and reduce viral replication capacity (27). Treatment-emergent R263K combined with additional integrase substitutions resulted in reduced susceptibility to dolutegravir versus R263K alone in both population and clonal phenotyping analyses. Viral clones with R263K and the integrase substitutions A49G, M50V, and V201I demonstrated reduced susceptibility to dolutegravir and integrase replication capacity compared with clones with R263K alone. Although A49G and M50V are not recognized as INSTI resistance-associated substitutions (27), treatment-emergent integrase substitutions at these positions or proximally at position 51 have been reported with other primary INSTI substitutions in participants on an INSTI-based combination regimen (9, 22, 28). Further investigation is needed to confirm the role of these positions in impacting INSTI susceptibility and viral fitness (22, 29). Both dolutegravir susceptibility and integrase replication capacity were further reduced in clones containing R263K and additional substitutions of E138T and S147G. This result is consistent with the increased evolutionary distance observed for clones with the known INSTI resistance-associated substitutions E138T and S147G compared with other clones evaluated at the same time point, suggesting that continued evolution in integrase occurred that led to reduced dolutegravir susceptibility and integrase replication capacity (27).

In the phylogenetic analysis, more diversity was seen in baseline sequences than those at PDVF. Within the clusters for each participant, there were subclusters with high bootstrap values that contained all sequences from the most recent time point. A substantial portion of clones sequenced at the most recent time point relative to PDVF were identical at the nucleotide level. In participant 1, the clustering of all week 36 and 136 clones containing R263K substitutions suggests a path of viral evolution. Further evidence that the R263K pathway allows the accumulation of secondary substitutions is demonstrated by the week 136 cluster from participant 1 (bootstrap value = 99%) in which 12 clones harbored A49G, M50V, V201I, and R263K and 4 clones with the greatest evolutionary distance had those substitutions plus E138T and S147G. These findings are consistent with viral populations showing attempted evolutionary divergence and drug pressure.

In an effort to understand the emerging HIV-1 resistance-associated substitutions at a molecular level, we and others previously described the development of HIV-1 intasome homology models containing G118R and R263K alone (9, 30). The resistance data presented here are consistent with and supported by our previous molecular analysis of the G118R and R263K models. Briefly, geometrical and hydrogen bond pattern changes at these amino acid locations modulate the relative positioning of the tDNA and vDNA catalytic loops, perturbing the geometry and trajectories of both substrates and leading to the observed resistance profiles and viral replication capacities. To further support our analysis, we developed 2 additional HIV-1 integrase homology models based on the cryo-EM intasome structure reported in the literature and the G118R HIV-1 intasome model described previously (9, 30). The first model contained the L74M V75A double mutant, and the second contained the L74M V75A G118R triple mutant. Addition of L74M and V75A occurred near the G118R mutation and the tDNA catalytic loop, resulting in the formation of a hydrophobic core by M74, A75, F121, L63, C65, and the side chain of E92 (Fig. 3). With additional mutations of L74M and V75A, a stronger hydrophobic core is formed just below the catalytic site that potentially restricts the flexibility of the tDNA catalytic loop and alters the geometry of that site.

Of the adolescents and children with treatment-emergent INSTI resistance in the P1093 study, 6 of 8 with resistance had the integrase substitution G118R or R263K, similar to resistance patterns observed in adults treated with dolutegravir (9, 22). The G118R integrase substitution had a greater impact on reduced dolutegravir susceptibility than R263K. The effect of both G118R and R263K on reduced dolutegravir susceptibility and integrase replication capacity was modulated by the presence of additional integrase substitutions. Homology models of the HIV-1 intasome complex provide mechanistic insights into how these unusual substitutions, which are not easily acquired by the virus during dolutegravir treatment, may contribute to the development of INSTI resistance. Overall, this study provides additional molecular and structural characterization of integrase to aid in the understanding of INSTI resistance mechanisms in antiretroviral-experienced populations.

## MATERIALS AND METHODS

### Study design.

P1093 is an ongoing, multicenter, open-label, noncomparative phase I/II study evaluating dolutegravir in infants, children, and adolescents with HIV-1 (ClinicalTrials.gov identifier NCT01302847). Participants were enrolled in 1 of 8 age- and formulation-defined cohorts: cohort I (adolescents ≥12 to <18 years), cohorts IIA and IIB (children ≥6 to <12 years), cohorts III and III-DT (children ≥2 to <6 years), cohorts IV and IV-DT (children ≥6 months to <2 years), and cohort V (infants ≥4 weeks to <6 months) (https://clinicaltrials.gov/ct2/show/NCT01302847; https://www.impaactnetwork.org/studies/p1093). Different dolutegravir formulations were evaluated across the cohorts, with cohorts I and IIA receiving film-coated tablets (FCTs); cohorts IIB, III, and IV receiving granules for suspension; and cohorts III-DT, IV-DT, and V receiving DT. This analysis describes INSTI resistance data from participants with available data through ≥24 weeks who met PDVF criteria across all recruited cohorts.

The P1093 study recruited participants with HIV-1 RNA levels of >1,000 copies/mL and a confirmed HIV-1 diagnosis. Participants with known exposure or resistance to INSTIs, active AIDS-defining opportunistic infections, or use of systemic interferon or chronic immunosuppressive agents were excluded. Participants received once-daily, weight-based dosing of dolutegravir administered in combination with an optimized background regimen for each participant, which was selected by the investigator according to pharmacokinetic sampling and baseline genotyping results and approved by the P1093 protocol team. Twice-daily dolutegravir dosing was provided if indicated, such as for combination dosing with NNRTIs (11). All optimized background regimens were required to contain ≥1 fully active drug and 1 additional drug other than dolutegravir based on genotypic testing at enrollment. Raltegravir, elvitegravir, bictegravir, or other INSTIs were not permitted in ART history or as background agents.

Protocol-defined virologic failure was defined as a confirmed decrease in plasma HIV-1 RNA of <1.0 log_10_ copies/mL at or after week 12 (unless HIV-1 RNA was <400 copies/mL) or confirmed HIV-1 RNA levels of >400 copies/mL at or after week 24 on 2 consecutive measurements at least 1 week but no more than 4 weeks apart. Participants could remain in the study and continue dolutegravir treatment after PDVF confirmation if the site investigators in consultation with the study team believed potential patient benefit existed. Participants who met PDVF criteria and remained in the study could either reoptimize their background regimen or continue with no changes.

The study was conducted at IMPAACT Network sites in accordance with the International Conference on Harmonization of Technical Requirements for Registration of Pharmaceuticals for Human Use Good Clinical Practice following the principles of the Declaration of Helsinki. The study protocol was reviewed and approved by local ethics committees or institutional review boards. Written informed consent was obtained from each participant’s parent or legal guardian, and assent was obtained from older children.

### Study assessments and data analyses.

For this interim analysis, all recruited participants with available data through ≥24 weeks of the study by 12 February 2019 across the age/formulation groups were evaluated for PDVF (10, 15–17, 31–33). Plasma HIV-1 RNA was measured at baseline and each available study visit using the Abbott RealTime HIV-1 assay (Abbott Molecular, Des Plaines, IL). If a participant met suspected virologic failure criteria, plasma HIV-1 RNA was evaluated ≥1 week but not >4 weeks from the date of suspected virologic failure to confirm PDVF. Plasma samples collected at screening and on or near PDVF were used for HIV-1 genotyping, which was conducted at the virology reference laboratory for IMPAACT (Seattle Children’s HIV Specialty Lab, Seattle, WA) and Monogram Biosciences (South San Francisco, CA), respectively. For genotyping performed at the IMPAACT reference laboratory, HIV-1 sequence analysis and viral subtyping were performed using the Stanford University HIV Drug Resistance Database (http://hivdb.stanford.edu). Blood samples for CD4^+^ cell count and frequency were assessed at baseline, upon entry, and at weeks 12, 24, 32, 40, and 48. An adherence questionnaire was administered that measured 3-day recall at each study visit. For additional adherence information, the study team conducted personal communications with the site principal investigator for all participants meeting PDVF criteria and reviewed the reason for study discontinuation from the end-of-study record.

### Clonal genotyping and phylogenetic analyses.

Clonal integrase genotyping and phenotyping for dolutegravir, raltegravir, and elvitegravir were performed at Monogram Biosciences. At least 8 clones were assessed at each time point. Phylogenetic analysis of 81 clonal and 13 population integrase amino acid sequences was performed. For each participant, identical nucleotide sequences were identified, and a single representative sequence was selected for inclusion in phylogenetic analyses. A maximum-likelihood tree was created using the IQ-TREE application with Jones-Taylor-Thornton plus Gamma modeling (34). Evolutionary distance branch support was determined using 1,000 bootstrap replicates. The maximum-likelihood tree used HXB2 as an outgroup.

### HIV integrase structural analyses.

The protein/substrate complexes involving amino acid changes at L74 and V75 were constructed as previously described using either the HIV-1 intasome structure (Protein Data Bank [PDB] ID 5U1C) or our G118R HIV-1 intasome homology model (9, 30). The wild-type complex was prepared using Maestro software (version 12.1.013; Schrödinger LLC, New York, NY) by adding hydrogens to all heavy atoms present (35, 36). The L74 and V75 residues in both wild-type HIV-1 integrase chains A and C were selected for mutation because both integrase chains are shown to be directly involved in the integration of either vDNA or tDNA substrates in the cryo-EM structure. The “Mutate Residue” function was first used to convert valine (V) to alanine (A) for the selected V75 residues, followed by conversion of leucine (L) to methionine (M) for the selected L74 residues. The “Select Rotamer” function was then used to select a conformation of the L74M mutant residue in chain A that best interacted with the surrounding residues while maintaining a conformation similar to the wild-type L74 amino acid side chain. A similar procedure was used for the L74M mutant residue in chain C. The protein/substrate complex containing the triple mutant L74M V75A G118R was constructed using the procedure described above using our previously generated HIV-1 intasome G118R homology model as the initial HIV-1 intasome structural template (9). The resulting coordinates were captured in Maestro software, and the hydrogens were removed from both proteins and substrates. The resulting complexes were exported as PDB files for further analysis, and images were created with the PyMOL molecular graphics system (version 1.7.6.6; Schrödinger LLC).

### Data availability.

Clonal and population sequences have been deposited in GenBank under accession numbers MZ568467 through MZ568547. Anonymized individual participant data and study documents can be requested for further research from www.clinicalstudydatarequest.com.

## References

[1] HHS Panel on Antiretroviral Therapy and Medical Management of Children Living with HIV. Guidelines for the use of antiretroviral agents in pediatric HIV infection. https://clinicalinfo.hiv.gov/sites/default/files/guidelines/documents/PedARV_GL.pdf. Accessed 20 September 2020.

[2] Buchanan AL, Montepiedra G, Sirois PA, Kammerer B, Garvie PA, Storm DS, Nichols SL. 2012. Barriers to medication adherence in HIV-infected children and youth based on self- and caregiver report. Pediatrics 129:e1244–e1251. 10.1542/peds.2011-1740. 22508915 PMC3340587

[3] Nachman S, Alvero C, Teppler H, Homony B, Rodgers AJ, Graham BL, Fenton T, Frenkel LM, Browning RS, Hazra R, Wiznia AA, Acosta E, Douglas S, Fry C, Kuryla S, Perdue L, Samson P, Spector S, Toye M, Tustin N, Watson S, Worrell C, Zheng N, IMPAACT 1066 study team. 2018. Safety and efficacy at 240 weeks of different raltegravir formulations in children with HIV-1: a phase 1/2 open label, non-randomised, multicentre trial. Lancet HIV 5:e715–e722. 10.1016/S2352-3018(18)30257-1. 30527329 PMC6537590

[4] Cahn P, Pozniak AL, Mingrone H, Shuldyakov A, Brites C, Andrade-Villanueva JF, Richmond G, Beltran Buendia C, Fourie J, Ramgopal M, Hagins D, Felizarta F, Madruga J, Reuter T, Newman T, Small CB, Lombaard J, Grinsztejn B, Dorey D, Underwood M, Griffith S, Min S, extended SAILING Study Team. 2013. Dolutegravir versus raltegravir in antiretroviral-experienced, integrase-inhibitor-naive adults with HIV: week 48 results from the randomised, double-blind, non-inferiority SAILING study. Lancet 382:700–708. 10.1016/S0140-6736(13)61221-0. 23830355

[5] Walmsley SL, Antela A, Clumeck N, Duiculescu D, Eberhard A, Gutiérrez F, Hocqueloux L, Maggiolo F, Sandkovsky U, Granier C, Pappa K, Wynne B, Min S, Nichols G. 2013. Dolutegravir plus abacavir-lamivudine for the treatment of HIV-1 infection. N Engl J Med 369:1807–1818. 10.1056/NEJMoa1215541. 24195548

[6] Raffi F, Jaeger H, Quiros-Roldan E, Albrecht H, Belonosova E, Gatell JM, Baril J-G, Domingo P, Brennan C, Almond S, Min S. 2013. Once-daily dolutegravir versus twice-daily raltegravir in antiretroviral-naive adults with HIV-1 infection (SPRING-2 study): 96 week results from a randomised, double-blind, non-inferiority trial. Lancet Infect Dis 13:927–935. 10.1016/S1473-3099(13)70257-3. 24074642

[7] Trottier B, Lake JE, Logue K, Brinson C, Santiago L, Brennan C, Koteff JA, Wynne B, Hopking J, Granier C, Aboud M. 2017. Dolutegravir/abacavir/lamivudine versus current ART in virally suppressed patients (STRIIVING): a 48-week, randomized, non-inferiority, open-label, phase IIIb study. Antivir Ther 22:295–305. 10.3851/IMP3166. 28401876

[8] Rhee S-Y, Grant PM, Tzou PL, Barrow G, Harrigan PR, Ioannidis JPA, Shafer RW. 2019. A systematic review of the genetic mechanisms of dolutegravir resistance. J Antimicrob Chemother 74:3135–3149. 10.1093/jac/dkz256. 31280314 PMC6798839

[9] Underwood M, Horton J, Nangle K, Hopking J, Smith K, Aboud M, Wynne B, Sievers J, Stewart EL, Wang R. 2021. Integrase inhibitor resistance mechanisms and structural characteristics in antiretroviral therapy-experienced, integrase inhibitor-naive adults with HIV-1 infection treated with dolutegravir plus two nucleoside reverse transcriptase inhibitors in the DAWNING study. Antimicrob Agents Chemother 66:e01643-21. 10.1128/AAC.01643-21. PMC8765460 34694877

[10] Viani RM, Alvero C, Fenton T, Acosta EP, Hazra R, Townley E, Steimers D, Min S, Wiznia A, for the P1093 Study Team. 2015. Safety, pharmacokinetics and efficacy of dolutegravir in treatment-experienced HIV-1 infected adolescents: forty-eight-week results from IMPAACT P1093. Pediatr Infect Dis J 34:1207–1213. 10.1097/INF.0000000000000848. 26244832 PMC4604048

[11] ViiV Healthcare. 2021. Tivicay package insert. ViiV Healthcare, Research Triangle Park, NC.

[12] ViiV Healthcare. 2020. ViiV Healthcare announces US FDA approval of the first-ever dispersible tablet formulation of dolutegravir, Tivicay PD, a once-daily treatment for children living with HIV. ViiV Healthcare, London, UK.

[13] ViiV Healthcare. 2021. ViiV Healthcare receives EU Marketing Authorisation for the first-ever dispersible-tablet formulation of dolutegravir, Tivicay, a treatment for children living with HIV in Europe. ViiV Healthcare, London, UK.

[14] ViiV Healthcare. 2021. Tivicay summary of product characteristics. ViiV Healthcare BV, Amersfoort, Netherlands.

[15] Vavro C, Ruel T, Wiznia A, Alvero C, Popson S, Hazra R, Townley E, Buchanan A, Stewart E, Palumbo P. 2020. Emergence of resistance in HIV-1 integrase following dolutegravir treatment in participants aged 4 weeks to <18 years: results from the IMPAACT 1093 study, abstr 26. *In* Abstr European Meeting on HIV & Hepatitis: Treatment Strategies & Antiviral Drug Resistance 2020.

[16] Viani RM, Ruel T, Alvero C, Fenton T, Acosta EP, Hazra R, Townley E, Palumbo P, Buchanan AM, Vavro C, Singh R, Graham B, Anthony P, George K, Wiznia A, Heckman B, Popson S, Sise T, Hergott K, Myers K, Rodriguez CA, Emmanuel PJ, Casey D, Wara D, Tilton N, Aziz M, McNichols M, Logan L, Sirisanthana V, Aurpibul L, Kosachunhanan N, Jensen J, Williams R, Qureshi T, Dobroszycki J, Huh H, Reinoso F, Rana S, Houston P, Mengistab M, Burchett SK, Karthas N, Kneut C, P1093 Study Team. 2020. Long-term safety and efficacy of dolutegravir in treatment-experienced adolescents with human immunodeficiency virus infection: results of the IMPAACT P1093 study. J Pediatric Infect Dis Soc 9:159–165. 10.1093/jpids/piy139. 30951600 PMC7192395

[17] Ruel T, Farhad M, Alvero C, Acosta EP, Singh R, George K, Montanez N, Popson S, Bartlett M, Dayton D, Anthony P, Buchanan A, Brothers C, Vavro C, Koech L, Vhembo T, Hazra R, Townley E, Wiznia A, IMPAACT P1093 Team. 2020. Twenty-four week safety, tolerability and efficacy of dolutegravir dispersible tablets in children 4 weeks to <6 years old with HIV-1: results from IMPAACT P1093, abstr PEB0293. *In* Abstr 23rd International AIDS Conference.

[18] Quashie PK, Mesplède T, Han Y-S, Veres T, Osman N, Hassounah S, Sloan RD, Xu H-T, Wainberg MA. 2013. Biochemical analysis of the role of G118R-linked dolutegravir drug resistance substitutions in HIV-1 integrase. Antimicrob Agents Chemother 57:6223–6235. 10.1128/AAC.01835-13. 24080645 PMC3837891

[19] Quashie PK, Oliviera M, Veres T, Osman N, Han Y-S, Hassounah S, Lie Y, Huang W, Mesplède T, Wainberg MA. 2015. Differential effects of the G118R, H51Y, and E138K resistance substitutions in different subtypes of HIV integrase. J Virol 89:3163–3175. 10.1128/JVI.03353-14. 25552724 PMC4337543

[20] Nachman S, Zheng N, Acosta EP, Teppler H, Homony B, Graham B, Fenton T, Xu X, Wenning L, Spector SA, Frenkel LM, Alvero C, Worrell C, Handelsman E, Wiznia A, Moultrie H, Kindra G, Sanders MA, Williams R, Jensen J, Acevedo M, Fabregas L, Jurgrau A, Foca M, Higgins A, Deville JG, Nielsen-Saines K, Carter MF, Swetnam J, Wilson J, Donnelly M, Akleh S, Rigaud M, Kaul A, Patel N, Gaur A, Utech LJ, Cardoso E, Moreira AM, Santos B, Bobat R, Mngqibisa R, Burey M, Abadi J, Rosenberg M, Luzuriaga K, Picard D, Pagano-Therrien J, Dittmer S, Ndiweni HN, International Maternal Pediatric Adolescent AIDS Clinical Trials (IMPAACT) P1066 Study Team, et al. 2014. Pharmacokinetics, safety, and 48-week efficacy of oral raltegravir in HIV-1-infected children aged 2 through 18 years. Clin Infect Dis 58:413–422. 10.1093/cid/cit696. 24145879 PMC3890333

[21] Nachman S, Alvero C, Acosta EP, Teppler H, Homony B, Graham B, Fenton T, Xu X, Rizk ML, Spector SA, Frenkel LM, Worrell C, Handelsman E, Wiznia A. 2015. Pharmacokinetics and 48-week safety and efficacy of raltegravir for oral suspension in human immunodeficiency virus type-1-infected children 4 weeks to 2 years of age. J Pediatric Infect Dis Soc 4:e76–e83. 10.1093/jpids/piu146. 26582887 PMC4681385

[22] Underwood MR, DeAnda F, Dorey D, Hightower K, Wang R, Griffith S, Horton J. 2015. Resistance post week 48 in ART-experienced, integrase inhibitor-naive subjects with dolutegravir (DTG) vs. raltegravir (RAL) in SAILING (ING111762), abstr 6. Abstr 13th European HIV & Hepatitis Workshop.

[23] ViiV Healthcare. 2021. Dovato package insert. ViiV Healthcare, Research Triangle Park, NC.

[24] ViiV Healthcare. 2021. Triumeq package insert. ViiV Healthcare, Research Triangle Park, NC.

[25] Gandhi M, Devi S, Bacchetti P, Chandy S, Heylen E, Phung N, Kuncze K, Okochi H, Kumar R, Kurpad AV, Ekstrand ML. 2019. Measuring adherence to antiretroviral therapy via hair concentrations in India. J Acquir Immune Defic Syndr 81:202–206. 10.1097/QAI.0000000000001993. 30865182 PMC6522327

[26] Phillips TK, Sinxadi P, Abrams EJ, Zerbe A, Orrell C, Hu N-C, Brittain K, Gomba Y, Norman J, Wiesner L, Myer L, Maartens G. 2019. A comparison of plasma efavirenz and tenofovir, dried blood spot tenofovir-diphosphate, and self-reported adherence to predict virologic suppression among South African women. J Acquir Immune Defic Syndr 81:311–318. 10.1097/QAI.0000000000002032. 30893125 PMC6565450

[27] Wensing AM, Calvez V, Ceccherini-Silberstein F, Charpentier C, Günthard HF, Paredes R, Shafer RW, Richman DD. 2019. 2019 update of the drug resistance mutations in HIV-1. Top Antivir Med 27:111–121. 31634862 PMC6892618

[28] Garrido C, Villacian J, Zahonero N, Pattery T, Garcia F, Gutierrez F, Caballero E, Van Houtte M, Soriano V, de Mendoza C. 2012. Broad phenotypic cross-resistance to elvitegravir in HIV-infected patients failing on raltegravir-containing regimens. Antimicrob Agents Chemother 56:2873–2878. 10.1128/AAC.06170-11. 22450969 PMC3370736

[29] Wares M, Mesplède T, Quashie PK, Osman N, Han Y, Wainberg MA. 2014. The M50I polymorphic substitution in association with the R263K mutation in HIV-1 subtype B integrase increases drug resistance but does not restore viral replicative fitness. Retrovirology 11:7. 10.1186/1742-4690-11-7. 24433497 PMC3898230

[30] Passos DO, Li M, Yang R, Rebensburg SV, Ghirlando R, Jeon Y, Shkriabai N, Kvaratskhelia M, Craigie R, Lyumkis D. 2017. Cryo-EM structures and atomic model of the HIV-1 strand transfer complex intasome. Science 355:89–92. 10.1126/science.aah5163. 28059769 PMC5508583

[31] Wiznia A, Alvero C, Fenton T, George K, Townley E, Hazra R, Graham B, Buchanan A, Vavro C, Viani R, P1093 Team. 2016. IMPAACT 1093: dolutegravir in 6- to 12-year-old HIV-infected children: 48-week results, abstr 816. *In* Abstr Conference on Retroviruses and Opportunistic Infections.

[32] Ruel T, Acosta EP, Singh R, Alvero C, Fenton T, George K, Townley E, Hazra R, Popson S, Buchanan AM, Brothers C, Vavro C, Wiznia A, IMPAACT P1093 Team. 2018. Pharmacokinetic and 4-week safety/efficacy of dolutegravir (S/GSK1349572) dispersible tablets in HIV-infected children aged 4 weeks to <6 years: results from IMPAACT P1093, abstr LBPEB023. *In* Abstr 12th International AIDS Conference.

[33] Ruel T, Acosta E, Singh R, Alvero C, George K, Popson S, Vavro C, Hazra R, Wiznia A, the P1093 Team. 2017. Dolutegravir pharmacokinetics, safety and efficacy in HIV+ children 2 to <6 years old, abstr 806. *In* Abstr Conference on Retroviruses and Opportunistic Infections.

[34] Trifinopoulos J, Nguyen L-T, von Haeseler A, Minh BQ. 2016. W-IQ-TREE: a fast online phylogenetic tool for maximum likelihood analysis. Nucleic Acids Res 44:W232–W235. 10.1093/nar/gkw256. 27084950 PMC4987875

[35] Schrödinger, LLC. 2019. Schrödinger release 2019–3: Maestro version 12.1.013. Schrödinger, LLC, New York, NY.

[36] Schrödinger, LLC. 2019. Schrödinger release 2019–3: MMshare version 4.7.013. Schrödinger, LLC, New York, NY.

