# Supplementary material for: Emergence of Resistance in HIV-1 Integrase with Dolutegravir Treatment in a Pediatric Population from the IMPAACT P1093 Study
Source: Antimicrob Agents Chemother. 2022 Jan 18;66(1):e01645-21. doi: 10.1128/AAC.01645-21 (PMC8765298; doi:10.1128/AAC.01645-21)
Supplement: Supplemental file 1 — Supplemental Text S1. Download AAC.01645-21-s0001.pdf, PDF file, 0.1 MB [file aac.01645-21-s0001.pdf]

Supplemental File S1

Emergence of Resistance in HIV-1 Integrase With Dolutegravir Treatment in a Pediatric Population From the IMPAACT P1093 Study

Cindy Vavro,[a] Theodore Ruel,[b] Andrew Wiznia,[c] Nicole Montañez,[d] Keith Nangle,[e] Joseph Horton,[e] Ann M. Buchanan,[a] Eugene L. Stewart,[f] Paul Palumbo[g]

[a]ViiV Healthcare, Research Triangle Park, NC, USA; [b]University of California San Francisco, San Francisco, CA, USA; [c]Jacobi Medical Center, Albert Einstein College of Medicine, Bronx, NY, USA; [d]FHI 360, Durham, NC, USA; [e]Parexel International, Durham, NC, USA; [f]GlaxoSmithKline, Upper Providence, PA, USA; [g]Geisel School of Medicine at Dartmouth, Lebanon, NH, USA

Background

HIV-1 integrase structural analyses described fully in the manuscript were carried out by either examination of the cryogenic electron microscopy (cryo-EM) structure of the HIV-1 intasome[1] or—in the case of dolutegravir (DTG)-bound HIV-1 INT—the cryo-EM structure of SIV intasome bound with DTG[2] downloaded from the Research Collaboratory for Structural Bioinformatics (protein data bank [PDB] ID: 5U1C and 6RWN, respectively) in PDB file format. Any mutant HIV-1 INT structures illustrated here were computationally derived from the cryo-EM HIV-1 intasome structure as described in the manuscript.

Scene 1 (0:04)

Cartoon and stick rendering of HIV-1 strand transfer complex intasome structure (also see Passos et al 2017,[1] Figure 1B).

The HIV-1 INT proteins are displayed in cartoon and colored magenta, while both the viral DNA (vDNA) and target DNA (tDNA) are in stick and colored orange. The intasome complex is composed of two pairs of dimeric HIV-1 INT proteins encompassing the 3’- and 5’-strands of both vDNA termini (shown vertically through the middle of the complex) and one double strand of the tDNA (horizontal near the bottom of the complex). The rendered green sphere is the catalytic Mg^{2+} and added for the purpose of identifying the catalytic pocket.

→ *Rotation and zoom in occurs to Scene 2*

Scene 2 (0:14)

View is perpendicular to the vDNA helical axis through to the catalytic Mg^{2+} and illustrating the catalytic/DTG-binding pocket. This perspective is orthogonal to the view of DTG and the HIV-1 INT catalytic pocket as shown in Figure 3A of the manuscript.

The surface of the HIV-1 INT catalytic site is rendered in transparent gray, with DTG oriented in that pocket, rendered in ball and stick, and colored by atom with carbons in white, oxygens in red, and nitrogens in blue. The nucleic acid components of the catalytic site are labeled as viral 5’-guanosine, viral 3’-cytidine, and viral 3’-adenosine, as are specific protein chain amino acids Y143, N144, and P145. Two Mg^{2+}s are rendered as green spheres and illustrate the binding of DTG to these Mg^{2+}s via its dual carbonyl oxygens.

→ Structure rotates to Scene 3

Scene 3 (0:20)

This view shows the vDNA and tDNA 3’-terminal nucleotides bound to **wild-type** G118. The cartoon and stick rendered protein is in magenta, and vDNA and tDNA substrates are shown in orange backbone with the terminal nucleotides rendered in stick and colored magenta. The green Mg^{2+} sphere and hydrogen bond interactions (yellow dashed lines) to the catalytic amino acid residues—D64 and D116—are shown with those residues depicted as sticks. The hydrophobic core of amino acids near the tDNA catalytic loop is displayed as sticks and colored by atom in magenta; this core is composed of amino acids L63, C65, L74, V75, and F121 and facilitates the position of the tDNA catalytic loop for proper strand transfer of the vDNA.

→ Structural changes are observed when wild-type G118 is replaced with R118 in Scene 4

Scene 4 (0:24)

The single-mutant R118 HIV-1 INT is colored orange and rendered in cartoon with key residues in the protein and substrates rendered as sticks. The color of the protein has been modified to highlight gross structural changes that occur when the wild-type G118 is replaced with R118 (colored by atom in cyan). Hydrogen bonds between the Mg^{2+} and catalytic amino acids—D64 and D116—remain (yellow dashed lines); however, an additional dual hydrogen bonding interaction with E92 (colored by atom in cyan) is observed and stabilizes the R118 substitution, facilitating an additional hydrogen bond with the 3’-hydroxy group of tDNA terminal thymidine. The hydrophobic core near the tDNA catalytic loop shown in the previous scene remains rendered here for reference.

→ Structural changes are observed when wild-type R118 is replaced with G118 and the hydrophobic core residue mutants M74 and A75 are present

Scene 5 (0:28)

This view illustrates the vDNA and tDNA 3’-terminal nucleotides bound to wild-type G118 with the additional amino acid mutants M74 and A75. The cartoon and stick rendered protein is in cyan, and vDNA and tDNA substrates are shown in orange backbone with the terminal nucleotides rendered in stick and colored magenta and orange, respectively. The green Mg^{2+} sphere and hydrogen bond interactions (yellow dashed lines) to the catalytic amino acid residues—D64 and D116—are shown with those residues depicted as sticks. The hydrophobic core of amino acids near the tDNA catalytic loop is displayed as sticks and colored by atom in pink; this core is composed of amino acids L63, C65, M74, A75, and F121; the two mutations in this region—M74 and A75—are clearly in proximity to the tDNA catalytic loop, directly interacting with F121 on that loop and affecting both loop and vDNA geometry. This perturbation leads to decreased ability of DTG to bind to the catalytic site but also is predicted to decrease the ability of the enzyme to efficiently perform strand transfer.

→ Structural changes are observed when wild-type G118 is replaced with R118 and the hydrophobic core residue mutants M74 and A75 are present

Scene 6 (0:32)

The single-mutant R118 HIV-1 INT is colored pink and rendered in cartoon with key residues in the protein and substrates rendered as sticks. The color of the protein has been modified to

highlight gross structural changes that occur when the wild-type G118 is replaced with R118 (colored by atom in cyan). Hydrogen bonds between the Mg^{2+} and catalytic amino acids—D64 and D116—remain (yellow dashed lines); however, an additional dual hydrogen bonding interaction with E92 (colored by atom in pink) is observed and stabilizes the R118 substitution, facilitating an additional hydrogen bond with the 3'-hydroxy group of tDNA terminal thymidine. The hydrophobic core of amino acids near the tDNA catalytic loop is displayed as sticks and colored by atom in pink; this core is composed of amino acids L63, C65, M74, A75, and F121; the two mutations in this region—M74 and A75—are clearly in proximity to the tDNA catalytic loop, directly interacting with F121 on that loop and affecting both loop and vDNA geometry. With the combination of the double mutant M74/A75 (via direct interaction with F121) and R118 (being an amino acid on the tDNA catalytic loop), the geometry of the tDNA catalytic loop is significantly perturbated such that binding of DTG and HIV-1 INT strand transfer efficiency is even further reduced compared with either the G118R or wild-type integrase.

→ *Structure rotates back to original Scene 1*

References

1. Passos DO, Li M, Yang R, Rebensburg SV, Ghirlando R, Jeon Y, Shkriabai N, Kvaratskhelia M, Craigie R, Lyumkis D. 2017. Cryo-EM structures and atomic model of the HIV-1 strand transfer complex intasome. Science 355:89–92.
2. Cook NJ, Li W, Berta D, Badaoui M, Ballandras-Colas A, Nans A, Kotecha A, Rosta E, Engelman AN, Cherepanov P. 2020. Structural basis of second-generation HIV integrase inhibitor action and viral resistance. Science 367:806–810.
